# A pareto strategy based on multi-objective optimal integration of distributed generation and compensation devices regarding weather and load fluctuations

**DOI:** 10.1038/s41598-024-61192-2

**Published:** 2024-05-07

**Authors:** Khaled Fettah, Talal Guia, Ahmed Salhi, Abir Betka, Abdelaziz Salah Saidi, Madjid Teguar, Enas Ali, Mohit Bajaj, Shir Ahmad Dost Mohammadi, Sherif S. M. Ghoneim

**Affiliations:** 1 Electrical Engineering Department, Laboratory (LNTDL), Hamma Lakhdar University of El Oued, 39000 El Oued, Algeria; 2 Electrical Engineering Department, Laboratory (LGEB), Mohamed Khider University of Biskra, b7000, Biskra, Algeria; 3 Electrical Engineering Department, Laboratory (LVCS), Hamma Lakhdar University of El Oued, 39000 El Oued, Algeria; 4 Electrical Engineering Department, College of Engineering, King Khalid University, 61421 Abha, Saudi Arabia; 5 Laboratoire Des Systèmes Électriques, École Nationale d’Ingénieurs de Tunis, Université de Tunis El Manar, Tunis, Tunisie; 6 Laboratoire de Recherche en Electrotechnique, Ecole Nationale Polytechnique, El-Harrach, 16200 Algiers, Algeria; 7 Centre of Research Impact and Outcome, Chitkara University Institute of Engineering and Technology, Chitkara University, Rajpura, Punjab 140401 India; 8 Department of Electrical Engineering, Graphic Era (Deemed to Be University), Dehradun, 248002 India; 9 Hourani Center for Applied Scientific Research, Al-Ahliyya Amman University, Amman, Jordan; 10 Graphic Era Hill University, Dehradun, 248002 India; 11 Department of Electrical and Electronics, Faculty of Engineering, Alberoni University, Kapisa, Afghanistan; 12 Department of Electrical Engineering, College of Engineering, Taif University, 21944 Taif, Saudi Arabia

**Keywords:** Multi-objective multi-verse optimization, Pareto-optimal solutions, The best compromise solution, Multi-objective jellyfish search, Multi-objective flower pollination, Multi-objective lichtenberg algorithm, Energy science and technology, Engineering, Mathematics and computing

## Abstract

In this study, we present a comprehensive optimization framework employing the Multi-Objective Multi-Verse Optimization (MOMVO) algorithm for the optimal integration of Distributed Generations (DGs) and Capacitor Banks (CBs) into electrical distribution networks. Designed with the dual objectives of minimizing energy losses and voltage deviations, this framework significantly enhances the operational efficiency and reliability of the network. Rigorous simulations on the standard IEEE 33-bus and IEEE 69-bus test systems underscore the effectiveness of the MOMVO algorithm, demonstrating up to a 47% reduction in energy losses and up to a 55% improvement in voltage stability. Comparative analysis highlights MOMVO's superiority in terms of convergence speed and solution quality over leading algorithms such as the Multi-Objective Jellyfish Search (MOJS), Multi-Objective Flower Pollination Algorithm (MOFPA), and Multi-Objective Lichtenberg Algorithm (MOLA). The efficacy of the study is particularly evident in the identification of the best compromise solutions using MOMVO. For the IEEE 33 network, the application of MOMVO led to a significant 47.58% reduction in daily energy loss and enhanced voltage profile stability from 0.89 to 0.94 pu. Additionally, it realized a 36.97% decrease in the annual cost of energy losses, highlighting substantial economic benefits. For the larger IEEE 69 network, MOMVO achieved a remarkable 50.15% reduction in energy loss and improved voltage profiles from 0.89 to 0.93 pu, accompanied by a 47.59% reduction in the annual cost of energy losses. These results not only confirm the robustness of the MOMVO algorithm in optimizing technical and economic efficiencies but also underline the potential of advanced optimization techniques in facilitating the sustainable integration of renewable energy resources into existing power infrastructures. This research significantly contributes to the field of electrical distribution network optimization, paving the way for future advancements in renewable energy integration and optimization techniques for enhanced system efficiency, reliability, and sustainability.

## Introduction

In the context of the rapidly progressing global electric power sector, there has been a notable upswing in overall societal electricity consumption within the past decade^1^. The conventional grid framework has traditionally seen the power sector adopt a centralized approach to energy generation and distribution. This entails the establishment of prominent power sources that are concentrated in specific regions, such as nuclear, hydropower, and coal-fired stations. These locations are chosen based on their capacity to satisfy the increasing demands of a growing population and industries^2^. After the establishment of these centralized power sources, the power sector initiates a wide-ranging infrastructure development, connecting different power sources through a complex network of transmission lines, transformers, and substations. The primary objective is to efficiently distribute the produced electricity across many regions, towns, and communities, guaranteeing a reliable and consistent power delivery^2^. Nevertheless, the traditional method faces constraints such as transmission losses over long distances, vulnerability to failures in centralized facilities, and environmental issues related to specific power sources such as coal^3,4^.

In recent years, there has been a substantial shift towards decentralized and renewable energy sources, such as solar and wind power. This transition is intended to address the constraints of traditional energy sources and promote a more resilient and sustainable energy system. The notion of distributed generation (DG) arose as a strategic approach in the 1980s to tackle the issues described earlier ^5^. DG has received substantial acclaim for its profound impact on altering the strategic planning and daily functioning of distribution networks ^6,7^. It proposes a significant change in thinking by promoting the incorporation of various, smaller power production sources into the grid. The deliberate incorporation of DG resources into distribution networks offers numerous benefits, such as significant improvements in power quality, decreased active power losses, optimization of voltage distribution, and resulting enhancements in the overall efficiency, flexibility, and economic feasibility of power network operations ^8,9^. The stability and operational efficiency of distribution networks are crucial in influencing the overall performance and effectiveness of the broader power system ^10^. The process of choosing the most suitable locations and sizes for distributed power generation resources has become a key area of focus in power grid design. Integrating DG technology is now crucial for establishing a robust, adaptable, and efficient power distribution network that is in line with current energy problems and goals.

The challenging process of establishing the appropriate placement and scale of distributed generation (DG) involves identifying the most favorable installation points and sizes that maximize advantages while meeting specified investment and operational criteria ^11,12^. Due to the increasing need for dependable power system operation, the issue of determining the location and size of distributed generation (DG) has progressed from solely focusing on reducing network losses to a complex optimization problem that takes into account various factors including voltage and current quality, as well as environmental concerns. In order to tackle this complex problem, a range of optimization techniques, such as quadratic programming methods and genetic algorithms, have been utilized. These strategies seek to address the inherent single-objective nature of the problem by converting it into a multi-objective format, thus enabling the generation of feasible solutions ^13^. Nevertheless, a notable obstacle occurs when applying these strategies, specifically in determining the suitable weighting variables. Implementing these weights in practice can be complex, as they impact the trade-offs between competing objectives and often lack explicit direction for selection.

Essentially, the process of optimizing the placement and size of distributed generation (DG) has progressed from a simple emphasis on reducing losses to a complex undertaking that involves finding a harmonious combination of technical, economic, and environmental factors. The ongoing changes in the energy sector require the development of more efficient approaches to deal with these intricacies. This is a crucial area of research in the field of power system planning and operation.

In addition, dealing with a wide range of planning models can be complex, and the choice of a suitable algorithm has a direct impact on the range of planning strategies^14,15^. At now, the field of algorithmic problem-solving mostly focuses on mathematical optimization and metaheuristic techniques^16^. The optimization methods in the field of distributed generators are constantly changing to fully exploit their potential. Among the methods used for this purpose, metaheuristic optimization techniques are highly prominent, providing a wide range of varied options. These techniques are widely used in solving optimization problems in several sectors, known for their superior efficiency in terms of execution durations compared to other optimization methods. Several research has focused on using multiobjective frameworks to improve the efficiency of distribution networks by optimizing the capacity and location of distributed generators. These studies utilize a wide range of strategies and techniques to accomplish these goals.

In recent research, various multi-objective optimization methodologies have been proposed. These approaches address a range of objectives and challenges. Notably^17^, introduces a strategy for optimizing systems with shunt capacitor banks to minimize costs, improve network reliability, and mitigate power losses and voltage deviations. In the presence of uncertain load demand^18^, utilizes the MOPSO optimization tool to devise a multi-objective plan for distribution systems. The incorporation of renewable energy sources is a key focus, as seen in^19^, which presents a comprehensive model encompassing photovoltaic arrays, wind turbines, and capacitor banks, targeting renewable generation, profit margins, emission reduction, power losses, voltage stability, and network security. ^20^ proposes an Advanced-PFNDMOPSO method for distributed generation optimization, integrating power loss reduction and voltage stability enhancement through fuzzy decision models. Further contributions include ^21^, which optimizes Dispersed Generators using multi-objective optimization on a radial distribution system, and ^22^, where the Whale Optimization Algorithm refines distributed generation and capacitor allocation, optimizing power losses and voltage deviations in an Indian rural network. ^23^ and ^24^ offer multi-objective frameworks for optimal DG placement and sizing, employing NSGA-III and fuzzy sets techniques, respectively. ^25^ adopts a fuzzy genetic algorithm for distributed generation and shunt capacitor allocation to reduce power losses. Additionally, ^26^ focuses on optimal allocation of various generation sources and capacitor banks considering uncertainties, while ^27^ formulates a novel objective function for renewable energy sources, employing multi-objective particle swarm optimization for validation. ^28^ investigates the benefits of integrating Distributed Generations (DGs) into radial distribution networks, enhancing performance through multi-objective optimization and Teaching–Learning-Based Optimization (TLBO). The MOPSO algorithm is leveraged in ^29^ for DG and capacitor bank allocation, outperforming genetic and particle swarm methods in reducing losses, enhancing voltage profiles, and lowering costs. Microgrid optimization is studied in ^30^, utilizing a fuzzy decision-maker and Pareto Front beam method for hourly performance optimization. The strategic placement of renewable DGs is emphasized in ^31^, while ^32^ proposes Improved Particle Swarm Optimization to optimize distribution system performance across various objectives. These endeavors collectively contribute to the advancement of multi-objective optimization techniques for enhancing the planning and operation of distribution systems.

Recent advancements in optimization techniques for electric power systems have emphasized the development and application of multi-objective optimization methods to enhance the integration and efficiency of various energy sources. ^33^ introduces the Adaptive Geometry Estimation-based Multi-objective Differential Evolution (AGE-MODE) method, tailored for optimizing power flow in hybrid systems that include thermal, wind, and solar energy sources, effectively addressing Multi-Objective Optimal Power Flow (MOOPF) challenges with multiple objectives. Concurrently, ^34–36^ discuss the Multi-Objective Search Group Algorithm (MOSGA), an evolution of the Search Group Algorithm (SGA) that incorporates elitist non-dominated sorting techniques and crowding distance strategies. This algorithm excels at identifying Pareto optimal solutions and optimizing power flow within systems powered by renewable energy by accounting for uncertainties in wind speed and solar irradiance, with its effectiveness validated through 25 case studies and IEEE systems. Additionally, ^37^ explores MOSGA's application in optimizing flat-plate solar collector systems, emphasizing improvements in thermal efficiency and cost reduction for nanofluids. Collectively, these studies highlight the potential of multi-objective optimization strategies in tackling the complex challenges of modern power systems, leading to significant benefits in economic, environmental, and technical aspects.

The objective of this work is twofold, emphasizing the development and application of a metaheuristic method named Multi-Objective Multi-Verse Optimization (MOMVO) for optimizing electrical distribution networks. The primary aim is to address the simultaneous minimization of daily energy losses and voltage profile deviations, responding adaptively to dynamic 24-h load conditions. This ambition crystallizes into a unified objective function (Obj1), which is meticulously expanded to incorporate the minimization of costs associated with energy losses and the power supplied by Photovoltaic and Reactive Energy Source Capacitor Banks (PVRES-CBs), forming a comprehensive objective model (Obj2). The approach's viability is assessed through simulations within distribution networks characterized by standard IEEE 33 and 69 nodes, conducting scenarios for PVRES-CBs allocation to attain the most significant reduction in the two targeted objective functions over a daily cycle. A distinctive feature of our approach is its consideration of both temperature and irradiation impacts on the PVRES and the network, diverging from previous methodologies that focused solely on irradiance. This broader perspective allows for a more accurate and realistic optimization of the distribution networks, accounting for the environmental variables that significantly affect the performance of PVRES.

The effectiveness of the proposed MOMVO algorithm is rigorously evaluated through a comparative analysis against three other prevalent optimization algorithms: Multi-Objective Jellyfish Search (MOJS), Multi-Objective Flower Pollination Algorithm (MOFPA), and Multi-Objective Lichtenberg Algorithm (MOLA). The comparison yields a Pareto front, which is crucial for decision-makers or power system operators faced with ambiguous or fuzzy objectives for each function. To assist in selecting the optimal operating point from the array of Pareto-optimal solutions, fuzzy logic theory is harnessed to derive fuzzy membership functions for each objective, aiming to identify the best non-dominated solution by maximizing the normalized sum of membership function values across all objectives. Furthermore, the study delves into the variations in the best compromise solutions derived from the aforementioned algorithms, providing valuable insights into the relative performances and suitability of these optimization strategies under varying network conditions.

In essence, this work strives to advance the optimization of electrical distribution networks through a nuanced, environmentally-responsive algorithm that not only enhances technical efficiency and cost-effectiveness but also fosters the sustainable integration of renewable energy sources. This research aspires to contribute substantially to the field of power system optimization, offering a robust framework for future innovations in the strategic integration of renewable energy and optimization techniques for improved power system efficiency, reliability, and sustainability.

As main contribution revolves around the introduction of a Multi-Objective Multi-Verse Optimization (MOMVO) approach, specifically designed for the simultaneous allocation of Photovoltaic and Reactive Power Compensation Equipment (PVRES-CB) in distribution networks. Notably, the inclusion of temperature as a new parameter enhances the accuracy of the electrical network analysis. This research thoroughly examines the performance of solar PVRES under real-world conditions, taking into account temperature variations and irradiance levels. The optimization process encompasses the consideration of two objectives functions and constraints across diverse 24-h load scenarios. Comparative analysis reveals that the MOMVO algorithm consistently outperforms the MOJS, MOFPA, and MOLA algorithms in effectively minimizing our defined objectives along the Pareto front. However, it is important to note that while MOMVO excels in most cases, it does not consistently yield the optimal compromise solution that meets all operational constraints. This occasional limitation can be attributed to the inherent randomness of these methods and their inability to comprehensively address dilemmas and achieve desired objectives across all scenarios.

Furthermore, this study showcases a significant reduction in losses, voltage deviations, and operational costs through the integration of a substantial number of Photovoltaic and Reactive Power Compensation Equipment (PVRES-CBs). This achievement underscores the remarkable success of this approach from both technical and practical perspectives. However, it is essential to acknowledge the economic implications, as the extensive utilization of PVRES and CBs comes with a substantial cost. This economic aspect must be thoroughly weighed and factored into any comprehensive study or analysis.

The subsequent sections of this paper delve into additional intricacies. Section "Mathematical model of MOMVO" expounds upon the mathematical framework underlying the Multi-Verse Optimization (MOMVO) algorithm, elucidating its core principles. Moving forward, Section “Allocation of photovoltaic renewable energy sources (PVRES) and capacitor banks (CB) for auxiliary service provision in distribution systems” delineates the mathematical intricacies of Photovoltaic and Reactive Energy Source (PVRES) allocation within distribution systems, providing a detailed exposition of the formulation. Section “Simulation and results” delves into an exhaustive examination of simulation outcomes derived from the MOMVO algorithm, specifically in the context of PVRES-CB allocation. This analysis encompasses diverse load scenarios spanning a 24-h timeframe. Lastly, Section “Conclusion” This study pioneers serves as the culmination of this study, encapsulating the final conclusions drawn from the research endeavor.

## Mathematical model of MOMVO

The realm of multi-objective optimization is significantly enriched by the advent of the Multi-Objective Multi-Verse Optimization (MOMVO) algorithm, which provides a nuanced approach to navigating the complexities inherent in optimizing multiple conflicting objectives simultaneously^38,39^. This advanced algorithm stands out for its capability to efficiently identify a spectrum of Pareto-optimal solutions. These solutions embody the principle of Pareto efficiency, where any attempt to improve one objective leads to the compromise of at least one other, emphasizing the necessity of balance in multi-objective optimization scenarios.

MOMVO innovates by envisioning each potential solution as a unique universe within an expansive multiverse, with the 'fittest' universes showcasing higher inflation rates, a metaphor for their optimization fitness. The algorithm employs cosmological concepts such as white holes, black holes, and wormholes to facilitate the flow of elements across these universes. This process not only encourages diversity within the solution space but also ensures a thorough exploration and exploitation of potential solutions, guiding the algorithm towards the Pareto front. This front represents an optimal trade-off curve where no solution can be considered superior to another based on all objectives, highlighting the relative efficiency of each solution within the defined multi-objective context.

The MOMVO algorithm's operation is akin to navigating a cosmos of possibilities, where each iteration brings it closer to the ideal set of solutions that best reconcile the competing objectives. This approach is particularly effective in complex decision-making environments that demand a delicate balance between different, often conflicting, criteria. The MOMVO algorithm's introduction into the field of optimization heralds a significant advancement, leveraging the metaphorical richness of cosmic phenomena to provide a fresh perspective on solving multi-objective problems. By expanding the metaheuristic optimization toolbox, MOMVO offers researchers and practitioners a sophisticated mechanism for making well-informed, balanced decisions across a broad spectrum of multi-objective optimization scenarios. This innovative approach not only enhances the theoretical framework of multi-objective optimization but also has practical implications for a wide range of applications, from engineering design to resource management and beyond, where complex trade-offs between multiple criteria are a common challenge.

The MVO algorithm relies on some principles during optimization:

- Each candidate solution represents a universe, and each variable is an object in that universe. Each solution is given an inflation rate corresponding the fitness function value.
- With higher inflation rate, the probability of having white holes’ increases, while the probability of having block holes’ decreases.
- While solutions with higher inflation rate often send objects through white holes, those with reduced inflation rate have a tendency to receive more objects via black holes.
- Through wormholes, the objects make random movements in the direction of the best universe.

### Mathematical model of the MVO can be described as follows

A) Initialization: MVO is a population based method, start the search with a number n of candidate solution, initialized randomly in the search space. These solutions are evaluated through a fitness function, and sorted from the best to the worst. Black and white holes are then used for exploring the search space and wormholes for exploitation.

(b) Exploration phase.

The MVO relies on the roulette wheel strategy to determine the universe that has the white hole, and uses Eq. (1) to move black and white holes between different universes:

1 $${x}_{i}^{j}=\left\{\begin{array}{l}{x}_{w}^{j} , \quad r<{NF}_{i}\\ {x}_{i}^{j} , \quad r\ge {NF}_{i}\end{array}\right.$$

where ${x}_{i}^{j}$ represents the ${j}^{th}$ variable of the candidate solution $i$. $w$ denotes the white hole Index selected randomly by the roulette wheel mechanism. $r$ is a random number uniformly distributed in the range [0, 1]. ${NF}_{i}$ indicates the normalized value of the fitness function of ${i}^{th}$ solution.

(c) Exploitation phase:

Utilizing the wormholes, the MVO makes local adjustments for each universe to perform an effective exploitation of the search space. The following formula describes how this process works:

2 $${{\varvec{x}}}_{{\varvec{i}}}^{{\varvec{j}}}\left\{\begin{array}{l}\left\{\begin{array}{l}{{\varvec{X}}}_{{\varvec{j}}}+TDR \times \left(\left({{\varvec{U}}{\varvec{B}}}_{{\varvec{j}}}-{{\varvec{L}}{\varvec{B}}}_{{\varvec{j}}}\right)\times {\varvec{r}}1+{{\varvec{L}}{\varvec{B}}}_{{\varvec{j}}}\right), r2<0.5\\ {{\varvec{X}}}_{{\varvec{j}}}-TDR \times \left(\left({{\varvec{U}}{\varvec{B}}}_{{\varvec{j}}}-{{\varvec{L}}{\varvec{B}}}_{{\varvec{j}}}\right)\times {\varvec{r}}1+{{\varvec{L}}{\varvec{B}}}_{{\varvec{j}}}\right), r2\ge 0.5\end{array}\right. , r3<WEP\\ {{\varvec{x}}}_{{\varvec{i}}}^{{\varvec{j}}} ,r3\ge WEP\end{array}\right.$$

${X}_{j}$ is the best solution found as far, ${UB}_{j}$ and ${LB}_{j}$ represent the upper and lower bounds of the search space. Three random numbers between [0,1] are chosen as $r1$, $r2$ and $r3$. The coefficients $TDR$ and $WEP$ are calculated as follows:

3 $$WEP={c}_{1}+t\times \left(\frac{{c}_{2}-{c}_{1}}{T}\right)$$

where ${c}_{1}$ and ${c}_{2}$ were fixed at 0.2 and 1 respectively.t denotes the current iteration and T the maximum iterations.

4 $$TDR=1-\frac{{t}^{1/p}}{{T}^{1/p}}$$

With $p$ is equal to 6.

### Implementation multi-objective multi-verse optimization for allocating PVRES-CBs problem

**Figure 1:**
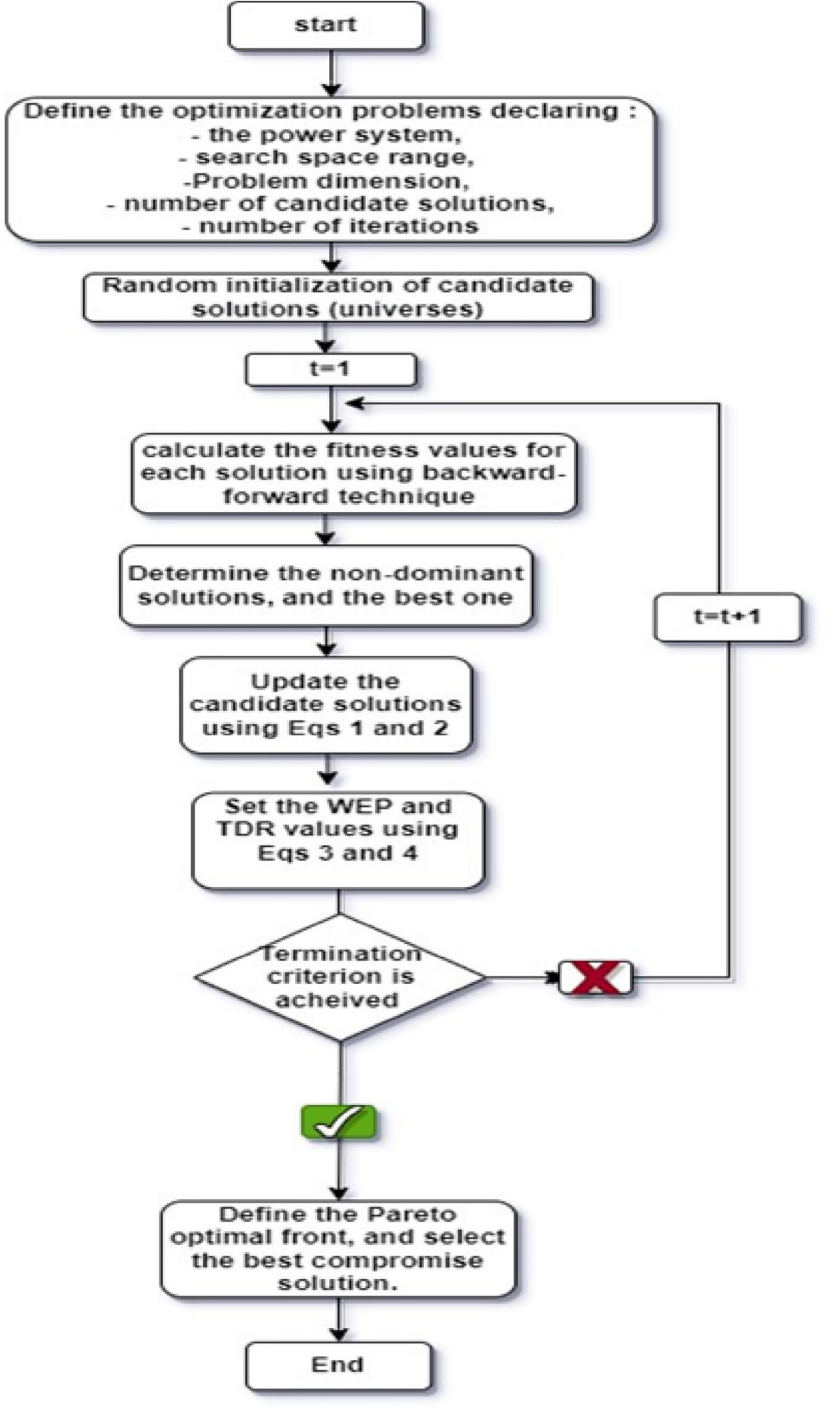
Flowchart of MOMVO.

The PVRES-CBs problem is solved using the MOMVO algorithm, in the aim to identify the set of parameters that are optimal and correspond to the minimal value of the considered objective function. The main process for applying the MOMVO algorithm to solve the PVRES-CBs problem is described in the following steps, and the suggested algorithm's flowchart is shown in Fig. 1.

Step 1. Definition of the optimization problem: Enter the data for the input power system. Declare of search space range, problem dimension, maximum number of candidate solutions, and number of iterations.

Step 2. Random initialization of candidate solutions.

Step 3. For each solution (universe), backward-forward technique is performed to calculate the fitness values (inflation rates) corresponding to both objective functions (Obj1 and Obj2).

Step 4. Determine the non-dominant solutions, and identify the best one. First, the crowding distance between the solutions in the archive is calculated. The best solution is then selected by using the roulette wheel technique.

Step 5. Normalize the solutions' fitness.

Step 6. Update the candidate solutions using Eqs. 1 and 2

Step 7. Verify if a solution variable is within the specified bounds, if not, we take the limits values and return it to the field.

Step 8. Set the $WEP$ and $TDR$ coefficients' values using Eqs. (3) and (4)

Step 9. Verify whether or not the termination criterion has been met. If not return to step 3, otherwise, go to step 10.

Step 10. Define the Pareto optimal front, and then using fuzzy set theory select the best compromise solution.

### Best compromise solution

After obtaining the Pareto-optimal front, fuzzy set theory is employed to identify the best compromise solution, which will generally satisfy all the different objectives.

(a). First, for each non-dominated solution $i$ and objective function $j$, a membership function value ${\varphi }_{i}^{j}$ is calculated, which indicates the level at which the original objective function has been achieved. It ranges from 0 to 1. 1 reflects a total satisfaction, whereas 0 represents an unsatisfactory result.

5 $${\varphi }_{i}^{j}=\left\{\begin{array}{l}\begin{array}{ll}1& \quad {\mathcal{F}}_{i}\le \end{array}{\mathcal{F}}_{i}^{min}\\ \begin{array}{ll}\frac{{\mathcal{F}}_{i}^{max}-{\mathcal{F}}_{i}}{{\mathcal{F}}_{i}^{max}-{\mathcal{F}}_{i}^{min}}&\quad {\mathcal{F}}_{i}^{min}{<\mathcal{F}}_{i}\end{array}\\ \begin{array}{ll}0& \quad { {\mathcal{F}}_{i}\ge \mathcal{F}}_{i}^{max}\end{array}\end{array}\right.<{\mathcal{F}}_{i}^{max}$$

${\mathcal{F}}_{i}^{max}$ and ${\mathcal{F}}_{i}^{min}$ represent the highest and lowest values of the i^th^ objective function found with all the non-dominated solutions.

(b) Then, a normalized membership value ${\varphi }^{j}$ is calculated, for each solution, using Eq

6 $${\varphi }^{j}=\frac{\sum_{i=1}^{{N}_{obj}}{\varphi }_{i}^{j}}{\sum_{i=1}^{{N}_{ds}}\sum_{i=1}^{{N}_{obj}}{\varphi }_{i}^{j}}$$

The best compromise solution could be the one that has the highest ${\varphi }^{j}$ value.

The MOMVO flowchart is illustrated in Fig. 1.

The selection of the Multi-Objective Multi-Verse Optimization (MOMVO) algorithm is justified by its unique approach to solving complex multi-objective optimization problems, specifically tailored to the integration of Distributed Generations (DGs) and compensation devices within distribution networks ^40,41^. The MOMVO algorithm is inspired by the multi-verse theory in physics, incorporating innovative mechanisms like black holes, white holes, and wormholes to navigate the search space effectively. This allows for a dynamic exploration and exploitation process, crucial for addressing the multifaceted objectives of reducing energy losses, voltage deviations, and minimizing costs associated with energy consumption and the power supplied by Photovoltaic and Reactive Energy Source Capacitor Banks (PVRES-CBs).

Our study employs MOMVO due to its proven effectiveness in generating well-distributed Pareto-optimal solutions, which are essential for capturing the trade-offs between competing objectives in a multi-objective optimization framework. The algorithm's ability to maintain a diverse set of solutions ensures comprehensive coverage of the solution space, leading to more informed and balanced decision-making. Furthermore, the inclusion of fuzzy set theory to select the best compromise solution from the Pareto front enhances the decision-making process by providing a systematic way to handle uncertainties and imprecision inherent in multi-objective optimization problems.

The choice of MOMVO over other algorithms is also validated through comparative analysis against alternatives like Multi-Objective Jellyfish Search (MOJS), Multi-Objective Flower Pollination Algorithm (MOFPA), and Multi-Objective Lichtenberg Algorithm (MOLA). The MOMVO algorithm's distinctive methodological features, combined with its demonstrated empirical success, provide a solid rationale for its selection. It offers a robust and effective tool for optimizing the complex interplay between technical and economic factors in the integration of DGs and compensation devices into distribution networks, aligning with the overarching goals of enhancing efficiency, reliability, and sustainability in power system operations.

## Allocation of photovoltaic renewable energy sources (PVRES) and capacitor banks (CB) for auxiliary service provision in distribution systems

### PVRES modeling

Generating power through PVRES modules is closely tied to meteorological factors, particularly solar radiation and ambient temperature^42^. The specific geographic location plays a pivotal role in shaping these factors. As a result, the evaluation of solar radiation conditions in a given area is typically carried out during the initial stages of effectively deploying PVRES panels ^43,44^. This process involves gathering historical data to compute both the standard hourly solar radiation and daily temperature. These calculated values are subsequently categorized into distinct phases, each of which is assigned predefined thresholds for solar radiation and temperature ^45,46^.

To optimize the attainment of maximum power output from a photovoltaic module, we employ a practical model as proposed by ^47^.

7 $${{\text{P}}}_{{\text{pv}}-{\text{max}}}={\text{FF}}\cdot \left({{\text{I}}}_{{\text{sc}}}\cdot \frac{{\text{G}}}{{{\text{G}}}_{{\text{ref}}}}\right)\cdot \left({{\text{V}}}_{{\text{oc}}}\cdot \frac{{\text{ln}}\left({{\text{P}}}_{1}\cdot {\text{G}}\right)}{{\text{ln}}\left({{\text{P}}}_{1}\cdot {{\text{G}}}_{{\text{ref}}}\right)}\cdot \frac{{{\text{T}}}_{{\text{jref}}}}{{{\text{T}}}_{{\text{j}}}}\right)$$

P(pv-max): The highest achievable power production of the photovoltaic (PV) system;

Isc: The current measured when the PV system is short-circuited;

G: The current level of solar irradiance (intensity of sunlight) at the specific time;

Gref: The standard reference solar irradiance;

Voc: The voltage measured when the PV system is open-circuited;

Tjref: The reference junction temperature of the PV system;

Tj: The actual junction temperature of the PV system at the given time.

The constant coefficient P1 can be determined using the following formula:

8 $${{\text{P}}}_{1}=\frac{{{\text{I}}}_{{\text{sc}}}}{{\text{G}}}$$

FF is the Filling factor given by:

9 $${\text{FF}}=\frac{{{\text{P}}}_{{\text{pvmax}}}}{{{\text{V}}}_{{\text{oc}}}\cdot {{\text{I}}}_{{\text{sc}}}}=\frac{{{\text{V}}}_{{\text{mpp}}}{{\text{I}}}_{{\text{mpp}}}}{{{\text{V}}}_{{\text{oc}}}{{\text{I}}}_{{\text{sc}}}}$$

Vmpp: Voltage corresponding to the Maximum Power Point (MPP) of the photovoltaic (PV) system.

Impp: Current corresponding to the Maximum Power Point (MPP) of the photovoltaic (PV) system.

### Allocation of photovoltaic renewable energy sources (Pvres) and capacitor banks (CB) for auxiliary service provision: constraints and objective

In allocating PVRES-CBs for auxiliary service provision within distribution systems, it is paramount to initially address the minimization of daily energy losses and voltage deviations. These objectives are amalgamated within a unified objective model (Obj1), aimed at simultaneous minimization, as detailed in Eq. (10).

10 $${\text{Obj1}} = {\text{Min}}({\text{ELD}},{\text{VDD}})$$

Where ELD represents the energy losses per day and VDD is the voltage deviation per day, which can be modeled as follows:

11 $${EL}_{\begin{array}{c}D\\ D\end{array}}={\sum }_{{\text{h}}=1}^{24} \left({\sum }_{{\text{br}}=1}^{{{\text{N}}}_{{\text{branches}}}} {\mathrm{ I }}_{{\text{br}}}^{ }{\left({\text{h}}\right) }^{2}\cdot {{\text{R}}}_{{\text{br}}}\right)$$

12 $${VD}_{\begin{array}{c}D\\ D\end{array}}={\sum }_{h=1}^{24}\left({\sum }_{j=1}^{buses}| 1-Vj|\right)$$

where N_branches_ is the number of distribution branches, I_br_ is the current flow through each distribution branch (br), R_br_ is the resistance of each distribution branch (br), N_buses_ is the number of buses, and V_j_ is the voltage magnitude at each distribution node (j)

Furthermore, within the context of assigning PVRES-CBs for auxiliary service provision in distribution systems, the reduction of costs related to energy losses and the power supplied by Photovoltaic and Reactive Energy Source Capacitor Banks (PVRES-CBs) is of utmost importance. These dual aims are encapsulated within a single objective model (Obj2), targeted for simultaneous minimization, as outlined in Eq. (13).

13 $${\text{Obj2}} = {\text{Min }}({\text{Closs}},{\text{TOC}})$$

where Closs denotes the yearly cost attributed to energy losses, and TOC signifies the cost of power supplied by Photovoltaic and Reactive Energy Source—Capacitor Banks (PVRES-CBs).

14 $${{\text{Closs}}={\text{Kp}}*\mathrm{ EL}}_{\begin{array}{c}D \\ D\end{array}}*{\text{Tf}}$$

15 $${\text{TOC}}={(\mathrm{Kp }* \sum }_{k=1}^{nPV}\mathrm{ PPV})+({\text{KQ}}*{\sum }_{k=1}^{ncb}\mathrm{ Qcb})$$

Kp, the annual cost of energy losses ($ /kwh).

Tf = 0.9*8760.

npv is the number of photovoltaic.

ncb is the number of Capacitor Banks.

Kp is the purchase cost ($ /KW).

PPV is the active power of the photovoltaic k.

KQ is the purchase cost ($ /KVAR).

Qcb is the reactive power of the capacitor bank k.

Hence, it is necessary to ensure that the actual power injection, both real and reactive, from PVRES-CB remains within the specified limits denoted by ${PPVRES}_{k\_max}$ and Qcb j_max, correspondingly, for every hour:

16 $$0 <{PPVRES}_{K}<{PPVRES}_{k\_max} k=1,.\dots ,nPV$$

17 $$0<{Q}_{{cb}_{j}}<{Q}_{{cb}_{j\_max}} j=1,\dots .,ncb$$

PPVRES refers to the real power injection from photovoltaic renewable energy sources (PVRES) into the grid, represented, while QCB represents the reactive power injection from capacitor banks (CB) into the system,

Furthermore, it is essential to ensure that the voltage at every distribution node and the current flowing through all distribution branches remain within the permissible limits throughout all hours, as stated in reference ^48^.

18 $${V}_{m\_min}<{V}_{m}<{V}_{m\_max} m=1,.\dots ,nB$$

19 $${{I}_{br} <I}_{{br}_{i\_max}} i=1,.\dots ,nbr$$

Here, ${V}_{m}$ represents the voltage at the bus *m*, ${V}_{m\_min}$ and ${V}_{m\_max}$ represent the lower and upper voltage bus limits, selected as 10% for all the network busses, and ${I}_{{br}_{i\_max}}$ is the thermal capacity limit of branch. These constraints are generally applied to all relevant hours and nodes or branches.

Moreover, as assumed in ^49^, a threshold on the PVs penetration is considered using the coefficient ${K}_{P}$, so that the PVs installed capacity ($\sum_{k\in nPV}{P}_{{PV}_{k}}$) is equal to 50% of the total active power demand in the system ${P}_{{D}_{m}}$

20 $$\sum_{k\in nPV}{P}_{{PV}_{k}}={K}_{P}\cdot \sum_{m\in nB}{P}_{{D}_{m}}$$

The inequality constraints in Eqs. (16) and (17) pertain to the control variables and are automatically managed through the MOMVO mechanism. However, additional attention is required to address the inequality constraints in Eqs. (18) and (20).

### Distribution network model

Typically, the resistance of the AC line is determined through the relationship:

21 $$R = r_{0} \cdot l$$

r0: Linear resistance per unit length [Ω/km];

l: Length of the line [m].

The line resistance allows determining the network one, denoted R_t_, as shown by the relation below:

22 $${\text{R}}_{{\text{t}}} = {\text{R}}\left[ {{1} + \alpha {25}\left( {{\text{t}}{-}{25}} \right)} \right]$$

Rt: Temperature-dependent resistance of the material at a specific temperature (t) [Ω];

R: Resistance of the material at a reference temperature (usually 25 °C) [Ω];

α25: Temperature coefficient of the resistance at the reference temperature (usually 25 °C);

t: Temperature for which the temperature-dependent resistance is calculated (in Celsius).

In this study, we adjust the resistance of all network buses (33, 69) to reflect real-world conditions, using 24 values of daily temperature as follows:

23 $${\text{R}}_{{{\text{ti}}}} = {\text{R }}[{1} + \alpha {25}\left( {{\text{t}}_{{\text{i}}} {-}{25}} \right)]$$

Rti: Resistance value during the i-th hour of the day, where i ranges from 1 to 24.

## Simulation and results

The MOMVO algorithm, as introduced in this study, has been strategically utilized to address and minimize two multifaceted objective functions that incorporate both technical performance and economic factors. This intricate optimization process takes into account the variability in the output from Photovoltaic and Reactive Power Compensation Equipment (PVRES), alongside the daily fluctuations in load demand. The effectiveness of this algorithm has been rigorously tested on two distinct electrical distribution systems: the IEEE 33 and IEEE 69 bus systems.

For the IEEE 33 bus system, the optimization strategy involved the allocation of three to six PVRES units coupled with nine capacitor banks, a configuration designed to balance the network's reactive power and enhance its voltage profile. The larger IEEE 69 bus system necessitated a more extensive integration of PVRES units five and ten units complemented by eighteen capacitor banks to effectively manage its higher load demand and more complex network structure.

The evaluation of each distribution system's performance was conducted through detailed simulations that utilized diverse operational profiles for PVRES, capacitor banks, and load variations spanning a 24-h period. The input parameters critical for this analysis, outlined in Table 1, encompass a range of factors including the capacity of PVRES units, the ratings of capacitor banks, and the hourly load profiles. The results derived from these simulations provide a comprehensive understanding of the algorithm's impact on the operational efficiency and economic viability of the distribution systems under study. The subsequent sections delve into a deeper analysis of these outcomes, offering insights into the algorithm's ability to enhance distribution system performance through optimized placement and sizing of PVRES and capacitor banks, thereby demonstrating the practical utility and adaptability of the MOMVO algorithm in optimizing distribution networks with varying scales and complexities.

**Table 1:** Daily energy loss and yearly cost of energy loss for different networks with minimum and maximum bus voltage.

Base case	IEEE 33	IEEE 69
Energy Loss (kWh)	3.5677e + 03	3.7970e + 03
Minimum bus voltage (pu) /12 h	0.9038	0.9092
Maximum bus voltage (pu)	1	1
The yearly cost of energy loss ($/year)	4.2191e + 06	4.4903e + 06

The study explores three distinct scenarios across both IEEE 33 and IEEE 69 networks, outlined as follows:

**In Scenario 1** involves a state analysis for each hour, incorporating power flow computations while accounting for temperature effects within the networks.

**In Scenario 2**, the proposed MOMVO algorithm is contrasted with (MOJS), (MOFPA), and (MOLA) approaches for the allocation of Photovoltaic and Reactive Power Compensation Equipment (PVRES) along with Capacitor Banks (CBs). This comparison aims to minimize the two designated objective functions, namely EqS. (10) and (13).

**In Scenario 3**, the application of the proposed MOMVO algorithm is expanded to encompass Photovoltaic and Reactive Power Compensation Equipment (PVRES) allocations. This extension involves doubling the number of PVRES units while keeping the quantity of capacitor banks consistent with that of Scenario 2. The primary aim remains the minimization of the two designated objective functions, namely Eqs. (10) and (13).

Both Scenarios 2 and 3 employ the MOMVO algorithm with 100 iterations and 30 search agents. Notably, in the first scenario, node voltages are restricted to 10% or lower of the nominal voltage.

**Figure 2:**
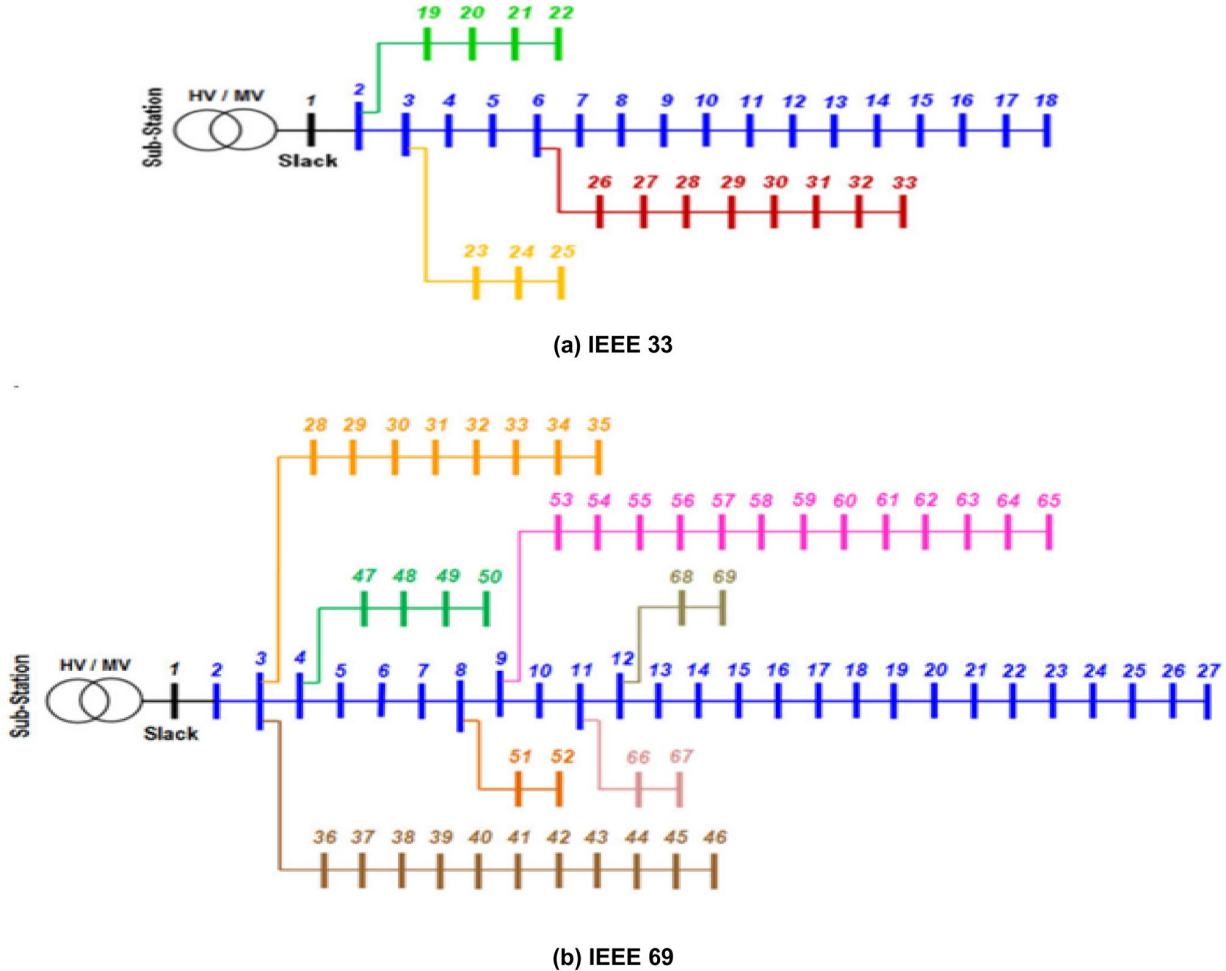
Configurations of the two distribution systems.

The effectiveness of the proposed MOMVO algorithm is evaluated across the IEEE 33 and IEEE 69 networks, which consist of 32 distribution sections and 33 nodes, and 68 distribution sections and 69 nodes, respectively. The technical specifications of branch and bus bar elements within the distribution networks are outlined in ^50^. Figure 2 provides a visual representation of the network diagrams for both systems, each operating at a nominal voltage of 12.66 kV.

**Figure 3:**
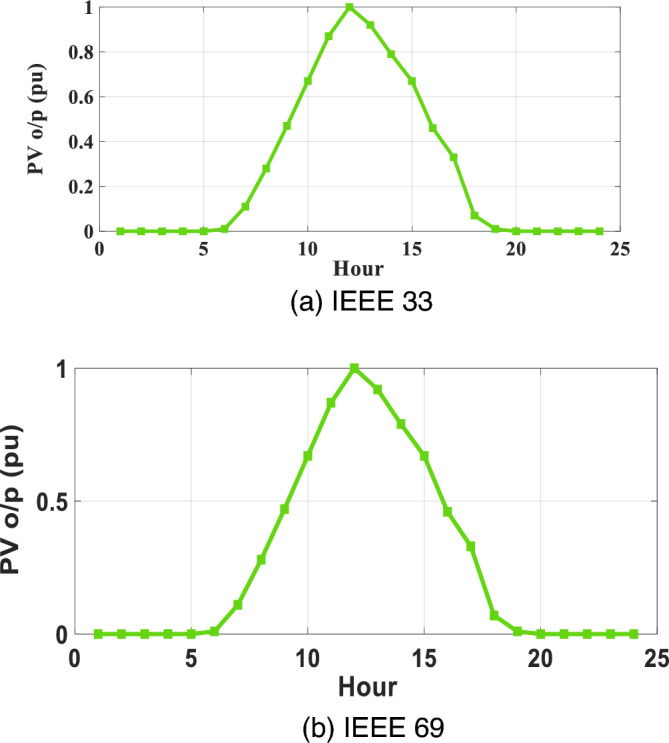
Annual daily average variations of PVRES with irradiance for the two networks.

**Figure 4:**
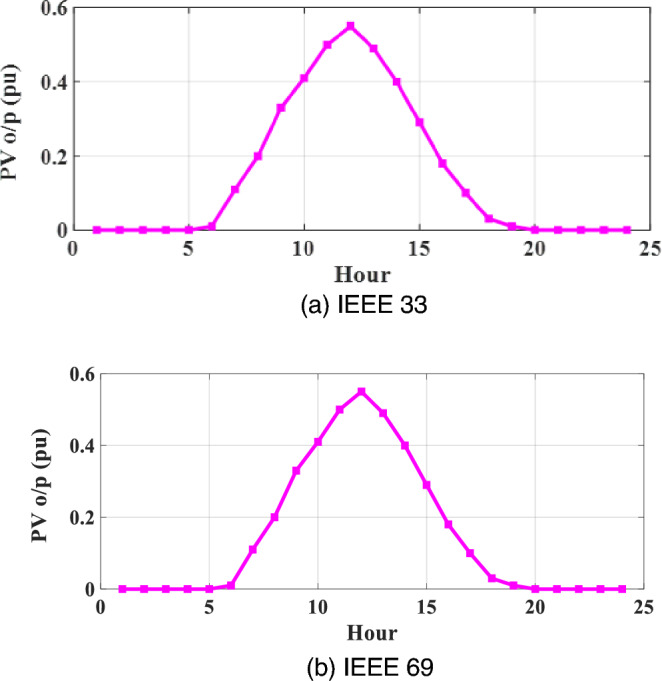
Annual daily average variations of PVRES with irradiance and temperature Effects for the two networks.

The LMSE laboratory, situated at Mohamed Khider University of Biskra in Algeria, possesses an extensive compilation of statistical data pertaining to the oscillation of Photovoltaic and Reactive Power Compensation Equipment (PVRES) in correlation with alterations in solar radiance and temperature for both IEEE 33 and IEEE 69 networks. The mean fluctuations of PVRES are meticulously outlined in the appendix (Table S1), playing a pivotal role in the formulation of Figs. 3 and 4.

**Figure 5:**
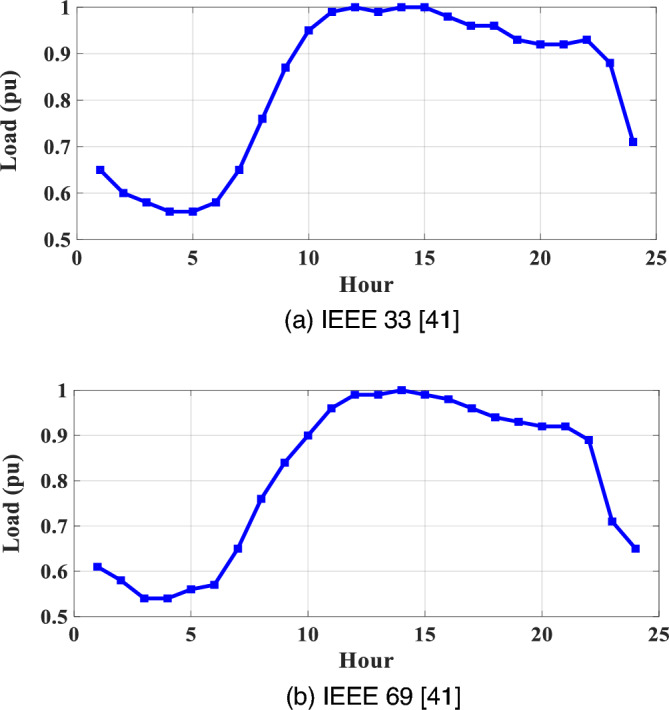
Annual daily average variations of for the two networks.

Figure 3a through b vividly depict the daily average deviations in PVRES power generation for the IEEE 33 and IEEE 69 networks, focusing exclusively on the impact of irradiance. In contrast, Fig. 4 provides an illustration of the average variations in PVRES over a 24-h timeframe per unit for the same networks, considering the joint influence of both irradiance and temperature. Additionally, Fig. 5 offers a visual representation of the load oscillations across these two systems.

The analysis presented in Figs. 3 and 4 of our study underscores the significant impact of temperature variations on the efficiency of photovoltaic modules, leading to notable fluctuations in the power output of Photovoltaic and Reactive Energy Source Capacitor Banks (PVRES-CBs) within the distribution networks. For the IEEE 33 network, the effect of temperature change is highlighted by a substantial decrease in power output, plummeting from 2100 kW at optimal conditions to 1155 kW as the temperature increases, reflecting a reduction to 27.5% of its maximum capacity. A similar trend is observed in the IEEE 69 network, where the power output drops from 1925 to 1058 kW, indicating a reduction to 26% of its maximum capacity under the influence of temperature variations.

Moreover, Fig. 5a and b provide a detailed visualization of the demand profile's peak periods, particularly evident around noon and lasting for approximately four hours. During these peak hours, the demand surges to its maximum, utilizing 100% of the loading capacity in both the IEEE 33 and IEEE 69 systems. This observation is crucial for understanding the dynamics of power consumption and the strain it places on the distribution networks, especially during periods of high demand.

These findings emphasize the necessity of incorporating temperature as a critical factor in the modeling and optimization of PVRES within distribution networks to ensure a reliable and efficient energy supply. The reduction in power output attributable to temperature elevations underscores the challenges in maintaining optimal operational efficiency and highlights the importance of developing robust optimization strategies, such as the proposed MOMVO algorithm, that can adapt to environmental changes and maintain the stability of the power distribution system.

Additionally, the peak demand observations suggest the need for strategic planning and allocation of PVRES-CBs to manage the load effectively during high-demand periods. By optimizing the placement and sizing of these resources, it is possible to mitigate the impact of peak demands on the distribution network's performance, ensuring a balanced and stable power supply throughout the day. This strategic approach not only enhances the operational efficiency of the distribution networks but also contributes to the overall resilience and reliability of the power system in the face of fluctuating demand and environmental conditions.

### Scenario 1

**Figure 6:**
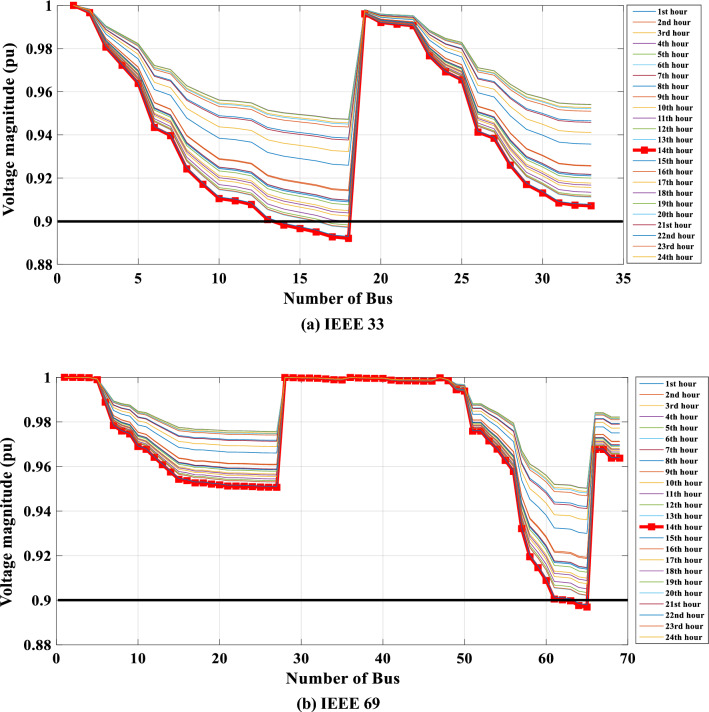
Hourly voltage profile over a 24-h period for the initial Scenario in the two networks.

In the given scenario, load flow computations are performed for each demand hour, taking into account the influence of temperature. As illustrated in Fig. 6a through b, the voltage distribution across all nodes of the distribution system, both for IEEE 33 and IEEE 69, is visually presented for each load hour. These voltage profiles consistently maintain values below the established minimum threshold, as evident from their distinct characteristics. It's notable that the voltage level drops to a minimum of 0.89 pu for both IEEE 33 and IEEE 69 during the peak consumption hour at hour 14. The impact of temperature on the network becomes even more evident through the lens of daily energy losses, meticulously outlined in Table 2, encompassing all networks.

**Table 2:** Daily energy loss and yearly cost of energy loss for all distribution systems with temperature effect.

	IEEE 33	IEEE 69
Energy Loss .e + 03 (kWh)	3.8313	4.0857
Minimum bus Voltage (pu)/14 h	0.8921	0.8969
Maximum bus voltage (pu)	1	1
The yearly cost of energy loss .e + 06 ($/year)	4.5308	4.8317

Furthermore, the yearly cost of energy loss is documented at 4.5308. e + 06 ($/year) for IEEE 33 and 4. 8317.e + 06 ($/year) for IEEE 6.

The data showcased in Table 2 underscores a notable increase in energy losses for both the IEEE 33 and IEEE 69 distribution networks, when subject to the impact of temperature fluctuations. Specifically, the energy losses registered at 3813 kWh for IEEE 33 and 4085.7 kWh for IEEE 69 exceed their initial baseline figures, which were 3567.7 kWh and 3797 kWh, respectively. This escalation translates to an approximate increase of 6.88% for the IEEE 33 network and 7.06% for the IEEE 69 network.

Furthermore, the annual costs associated with these energy losses were calculated to be $4.5308 million for IEEE 33 and $4.8317 million for IEEE 69. When compared to the baseline values of $4.2191 million for IEEE 33 and $4.4903 million for IEEE 69, these figures represent an increase of about 7.38% and 7.60% respectively. This quantifiable rise in both energy losses and associated costs clearly delineates the significant influence of temperature variations on the operational efficiency of these networks.

Such findings not only underscore the critical need to incorporate environmental factors like temperature into the planning and optimization of distribution networks but also highlight the potential for enhanced operational and financial efficiency through the adoption of optimized energy management strategies. This emphasizes the importance of developing and implementing robust solutions capable of adapting to environmental variations to maintain the stability and efficiency of power distribution systems.

### Senario 2

In this scenario, the Multi-Objective Multi-Verse Optimization (MOMVO) method is evaluated against other optimization techniques—namely the Multiobjective Flower Pollenation Algorithm (MFPA), Multiobjective Lion Algorithm (MOLA), and Multiobjective Jellyfish Search (MOJS)—with a focus on optimizing the allocation of Photovoltaic and Reactive Energy Source Capacitor Banks (PVRES-CBs) across distribution networks. The comparative analysis, anchored by objective functions encapsulated in Eq. 10 and Eq. 13, takes into account various configurations of PVRES allocations and capacitor banks (CBs), facilitating a comprehensive evaluation of each method's efficacy.

Table 3 delineates the best compromise solutions identified for minimizing energy losses and voltage deviations (the first objective) and reducing the annual cost of energy losses alongside the cost of power supplied by distributed generators (DGs) (the second objective), for both the IEEE 33 and IEEE 69 networks. The MOMVO method's application to the IEEE 33 network resulted in a notable 47.58% reduction in daily energy loss (from 3831.3 to 2008.1 kWh/day) and an improvement in voltage stability (from 0.89 to 0.94 pu). Similarly, for the second objective, a significant 36.97% decrease in the annual cost of energy loss (from $4.5308 million to $2.8556 million) was observed, alongside achieving a cost of $39,146/year for power supplied by DGs.

**Table 3:** Optimal location and size of PVRES and CB with the best compromise solution of energy losses, Minimum bus Voltage (pu), and the yearly cost of energy losses, and the cost of power supplied by Photovoltaic and Reactive Energy Source—Capacitor Banks (PVRES-CBs) obtained for scenario 2 for all distribution network.

Comparison Criteria	Optimal Placement and Sizing of Photovoltaic (PV) and Capacitor Banks (CB)						Minimum bus voltage (pu)				Best compromise solution		Best compromise solution	
	–		3 PVRES and 9 CB for IEEE 33		5 PVRES and 18 CB for IEEE 69		IEEE 33		IEEE 69		IEEE 33	IEEE 33	IEEE 69	IEEE 69
			Obj1	Obj2	Obj1	Obj2	Obj1	Obj 2	Obj1	Obj2	Obj1	Obj2	Obj1	Obj2
Base case	–	0.90	0.90	–	–	–								
Scenario 1	–	0.89	0.89											
Techniques	MOFPA	PV buses	12 16 32	12 16 31	19 59 60 61 64	19 61 62 63 64	0.941	0.91	0.932	0.919	2.0876e + 03 0.6118	2.7109e + 06 3.9196e + 04	2.2457e + 03 0.2150	2.8852e + 06 3.5902e + 04
		CB buses	13–14–15–17–31–32–33	14–18–31–32–33	19 20 21 22 24 25 26 27 57 58 59 60 61 62 64 65	24 61 63 64 65								
		CB Size	150 –150 150 –150 150 –150 150	50–50–150–150–50	100 –150 – 150 150 –100 – 100 150 –150 – 150 50 –150 – 100 150 –150 – 150 150	50 150 50 100								
	MOLA	PV buses	17 30 32	12 15 31	60 62 63 64 67	13 15 22 60 61	0.945	0.919	0.934	0.917	2.3275e + 03 0.5655	2.6514e + 06 3.9221e + 04	2.7973e + 03 0.1477	2.9901e + 06 3.5952e + 04
		CB buses	13– 14– 15 16– 17– 18 31– 32– 33	14– 18 – 31– 32– 33	19 20 21 22 23 24 25 26 27 57 58 59 60 61 62 63 64 65	24–59– 61– 64								
		CB Size	150 -100 150 –150 100 –100 150 –100 150	50 –50 –150 100 – 150	150 150 150 150 150 150 150 150 150 150 150 150 150 150 150 150 150 150	50 150 150 100								
	MJS	PV buses	29 30 32	14 16 31	3 12 42 58 59	15 23 60 63 64	0.947	0.923	0.926	0.924	2.5748e + 03 0.5074	2.5511e + 06 3.9271e + 04	2.7153e + 03 0.2068	2.5494e + 06 3.6102e + 04
		CB buses	13– 14– 15 16– 17– 18 31– 32– 33	15–31– 32– 33	19 20 21 22 24 25 26 27 59 60 61 62 63 64 65	24 59 60 61 64 65								
		CB Size	150 –150 150 –150 –50 150 –150 150 –150	150 –100 100 –150	100 100 150 100 150 50 150 150 150 100 150 150 150 150 150	150 100 50 150 150 100								
	MOMVO	PV buses	12 15 31	12 16 31	20 60 61 63 64	59 60 61 63 64	0.943	0.914	0.933	0.924	2.0081e + 03 0.6623	2.8556e + 06 3.9146e + 04	2.0364e + 03 0.2397	2.5320e + 06 3.6002e + 04
		CB buses	13– 14– 15 16– 18 – 31 32– 33	15–31– 32	20 21 22 23 24 27 58 59 60 61 62 63 64 65	60 61 64 65								
		CB Size	150– 150 100 – 50 50 – 150 150 –150	50 –150 –150	100 150 150 150 150 100 150 150 50 150 150 50 150 150	50 200 150 150								

For the IEEE 69 network, MOMVO showcased a 50.15% reduction in energy loss (from 4085.7 to 2036.4 kWh/day) and an improvement in voltage profiles (from 0.89 to 0.93 pu). Furthermore, there was a 47.59% decrease in the annual cost of energy loss (from $4.8317 million to $2.5320 million), with the cost for power supplied by DGs recorded at $36,002/year.

**Figure 7:**
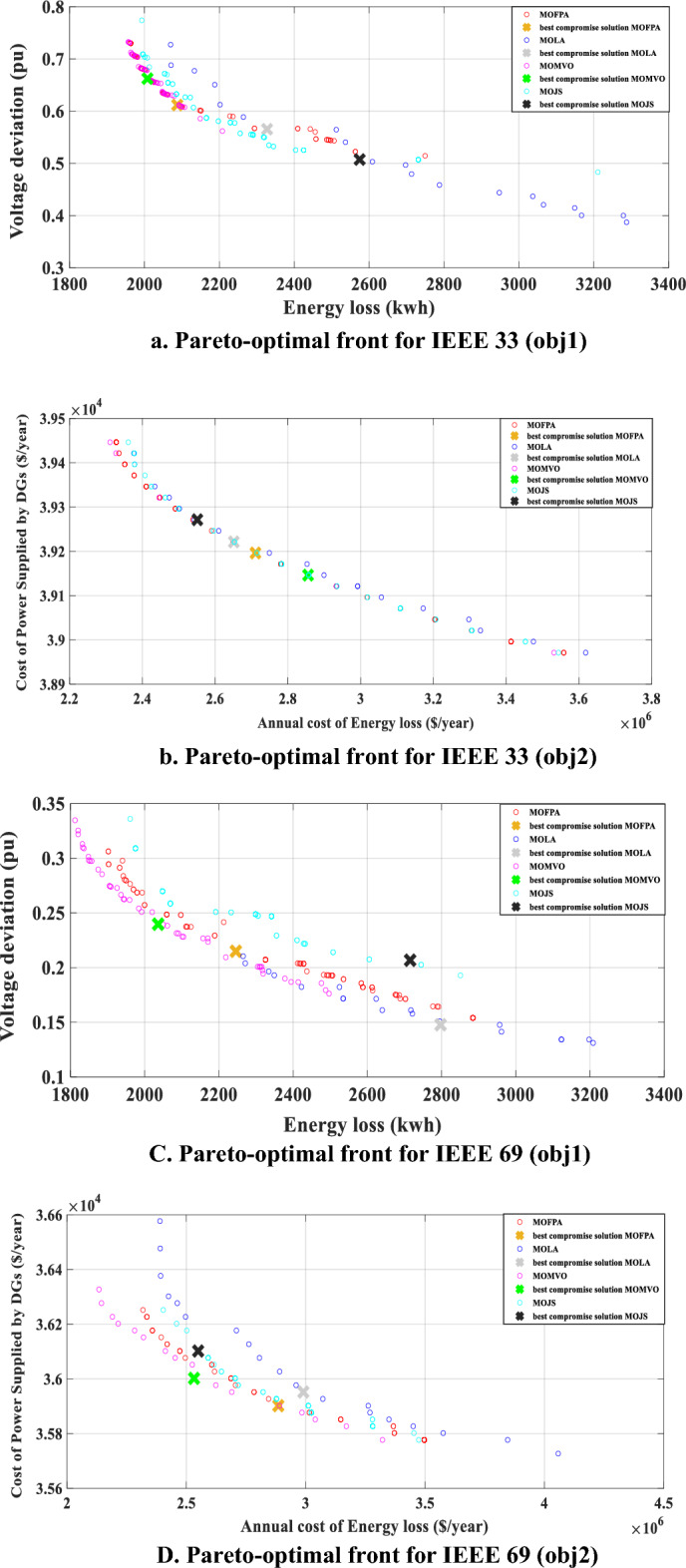
Pareto-Optimal Front Using MOMVO, MOFPA, MOLA, and MOJS for the Best Solution in the Second Scenario Across All Distribution Systems.

These results underscore the MOMVO method's superior reliability and efficacy in optimizing network performance compared to the MOJS, MFPA, and MOLA methods. The diverse solutions represented on the Pareto front, as illustrated in Fig. 7a–d for both IEEE networks, highlight the effectiveness of MOMVO's diversity-preserving mechanisms throughout the optimization process. The graphical representations of the Pareto solutions clearly demonstrate MOMVO's enhanced performance over the comparative algorithms, signifying its capability to achieve optimal convergence and significantly improve network performance through the strategic allocation of PVRES-CBs.

The MOMVO method not only excels in minimizing the specified objectives but also enhances the overall operational efficiency of the distribution networks. Its success in the comparative analysis suggests its potential as a robust and reliable tool for distribution network optimization, offering substantial improvements over existing methodologies in addressing the complexities of modern electrical distribution systems.

### Scenario 3

In this analytical scenario, our investigation zeroes in on the performance evaluation of the Multi-Objective Multi-Verse Optimization (MOMVO) algorithm in juxtaposition with three other esteemed optimization methods: Multi-Objective Jellyfish Search (MOJS), Multi-Objective Lion Algorithm (MOLA), and Multi-Objective Flower Pollination Algorithm (MOFPA). This comparative study is motivated by the overarching aim to optimize two pivotal objective functions, as delineated in Eq. (10) and Eq. (13), across various allocations of Photovoltaic and Reactive Energy Source Capacitor Banks (PVRES-CBs) within the distribution networks of IEEE 33 and IEEE 69.

Table 4 meticulously catalogues the outcomes, presenting a holistic view of the most balanced solutions concerning energy losses and voltage deviations (as the primary objective) along with the annual financial burdens stemming from energy losses and the costs incurred from the power supplied by Distributed Generators (DGs) as the secondary objective. Specifically, within the IEEE 33 network, the implementation of the MOMVO strategy heralds a profound reduction in energy loss, showcasing a decrease of 54.34% from an initial value of 3831.3 kWh/day to a mere 1749.2 kWh/day. This significant reduction is complemented by an improvement in the voltage deviations index to 0.60 and an elevation of voltage profiles from 0.89 to 0.94 pu, perfectly aligning with the primary objective. Furthermore, the secondary objective witnesses a considerable decrease of 53.20% in the annual cost of energy loss, plummeting from $4.5308 million in the baseline scenario to $2.1204 million. Additionally, a noteworthy achievement of $78,243 is recorded for the cost associated with power supplied by DGs.

**Table 4:** Optimal location and size of PVRES and CB with the best compromise solution of energy losses, Minimum bus Voltage (pu), and the yearly cost of energy losses, and the cost of power supplied by Photovoltaic and Reactive Energy Source—Capacitor Banks (PVRES-CBs) obtained for scenario 3 for all distribution network.

Comparison Criteria	Optimal Placement and Sizing of Photovoltaic (PV) and Capacitor Banks (CB)						Minimum bus Voltage (pu)				Best compromise solution		Best compromose solution	
	–		6 PVRES and 9 CB for IEEE 33		10 PVRES and 18 CB for IEEE 69		IEEE 33		IEEE 69		IEEE 33	IEEE 33	IEEE 69	IEEE 69
			Obj1	Obj2	Obj1	Obj2	Obj1	Obj2	Obj1	Obj2	Obj1	Obj2	Obj1	Obj 2
Base case	–						0.90		0.90		–	–	–	
Scenario 1	–						0.89		0.89					
Techniques	MOFPA	PV buses	8 12 16 24 29 32	8 12 16 28 30 31	7 9 14 57 59 60 61 62 64 68	9 10 19 20 21 59 60 61 63 64	0.942	0.919	0.933	0.921	1.9767e + 03 0.5950	2.3548e + 06 7.8143e + 04	2.3684e + 03 0.1613	2.3997e + 06 7.1729e + 04
		CB buses	13 14 15 16 17 18 31 33	17 18 31 32	20 21 22 24 25 26 27 57 58 59 60 61 63 64 65	20 21 57 59 61 64 65								
		CB Size	150 150 150 50 150 150 150 150	50 50 150 150	150 150 150 150 100 150 150 150 150 150 150 150 100 150 150	50 50 50 150 100 50 100								
	MOLA	PV buses	15 26 29 30 31 32	6 8 12 16 29 31	48 52 58 59 60 61 62 63 64 66	5 7 17 32 56 59 60 62 63 64	0.947	0.918	0.93	0.918	2.1990e + 03 0.5046	2.3074e + 06 7.8168e + 04	1.9993e + 03 0.2105	2.6917e + 06 7.1629e + 04
		CB buses	13 14 15 16 17 18 31 32 33	13 14 31 32 33	19 20 21 22 23 24 25 26 27 57 58 59 60 61 62 63 64 65	57 59 60 61 62								
		CB Size	150 150 150 150 150 100 150 150 150	100 50 100 100 100	150 150 150 150 150 150 150 150 150 150 150 150 150 150 150 150 150 150	50 50 50 100 100								
	MJS	PV buses	8 11 14 24 30 31	11 13 16 28 29 31	4 6 15 59 60 62 63 64 68 69	16 20 52 53 56 58 60 63 67 68	0.939	0.919	0.928	0.919	1.6641e + 03 0.7356	2.3632e + 06 7.8143e + 04	2.1012e + 03 0.2310	2.7209e + 06 7.1704e + 04
		CB buses	13 15 16 17 18 31 32 33	18 31 32 33	20 21 22 23 24 25 26 27 58 60 61 62 63 64 65	20 25 26 64 65								
		CB Size	150 150 100 50 50 150 150 150	100 150 50 100	150 50 150 50 150 50,150 150 100 50 100 150 150 150 150	150 50 50 150 100								
	MOMVO	PV buses	8 13 17 28 30 31	7 12 16 28 29 31	18 19 57 58 59 60 61 62 63 64	7 21 23 57 58 59 60 62 63 64	0.947	0.92	0.93	0.923	1.7492e + 03 0.6095	2.1204e + 06 7.8243e + 04	1.5507e + 03 0.2897	2.2730e + 06 7.1679e + 04
		CB buses	13 14 15 16 18 31 32 33	14 31 32 33	20 21 22 24 27 57 58 61 62 64 65	60 64 65								
		CB Size	150 150 150 150 100 150 150 150	150 200 200 50	50 150 150 50 150 150 100 150 150 150 150	150 100 150								

Turning our attention to the IEEE 69 network, the application of the MOMVO approach results in a stellar 62.04% diminution in energy loss, effectively bringing it down from the preliminary figure of 4085.7 kWh/day to 1550.7 kWh/day. This reduction is supported by a voltage index of 0.2897 and a noticeable improvement in voltage profile variations, escalating from 0.89 to 0.93 pu, in harmony with the primary objective. In relation to the secondary objective, the annual cost of energy loss witnesses a substantial decline of 52.95%, from an initial $4.8317 million to $2.2730 million, with the cost of power supplied by DGs marked at $71,679.

The data encapsulated in Table 4 unequivocally attests to the superior performance of the MOMVO method over the MOJS, MOLA, and MOFPA techniques, highlighting its increased reliability and effectiveness in optimizing the placement and sizing of PVRES units and CBs to meet the predefined objectives.

**Figure 8:**
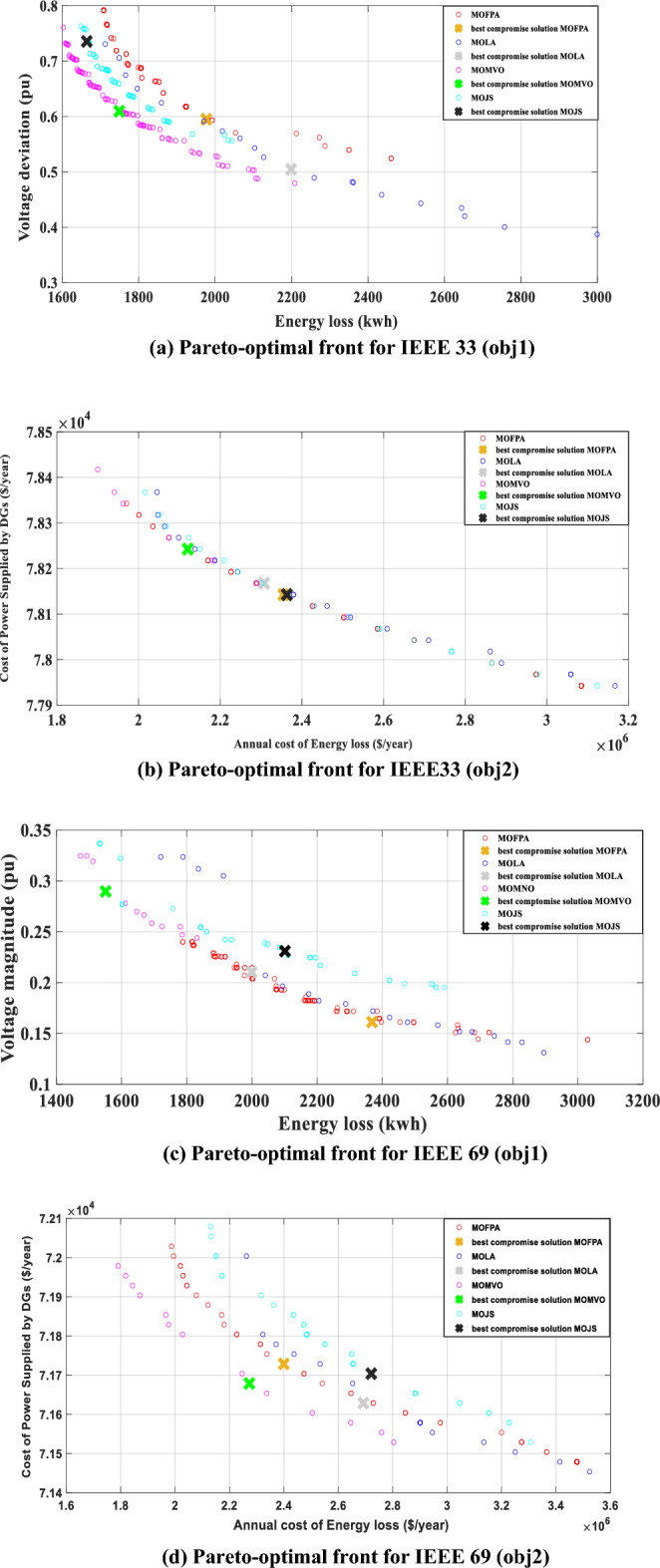
Pareto-Optimal Front Using MOMVO, MOFPA, MOLA, and MOJS for the Best Solution in the third Scenario Across All Distribution Systems.

This comparative analysis is further enriched by the convergence behaviors depicted in Fig. 8a-c for both IEEE networks, reinforcing the observations from scenario two (as illustrated in Fig. 7). Remarkably, Fig. 8 accentuates the MOMVO algorithm's superior convergence characteristics in determining the optimal positions and dimensions of PVRES and CBs, thereby achieving the objectives and significantly enhancing the operational efficiency of the distribution networks under study.

**Figure 9:**
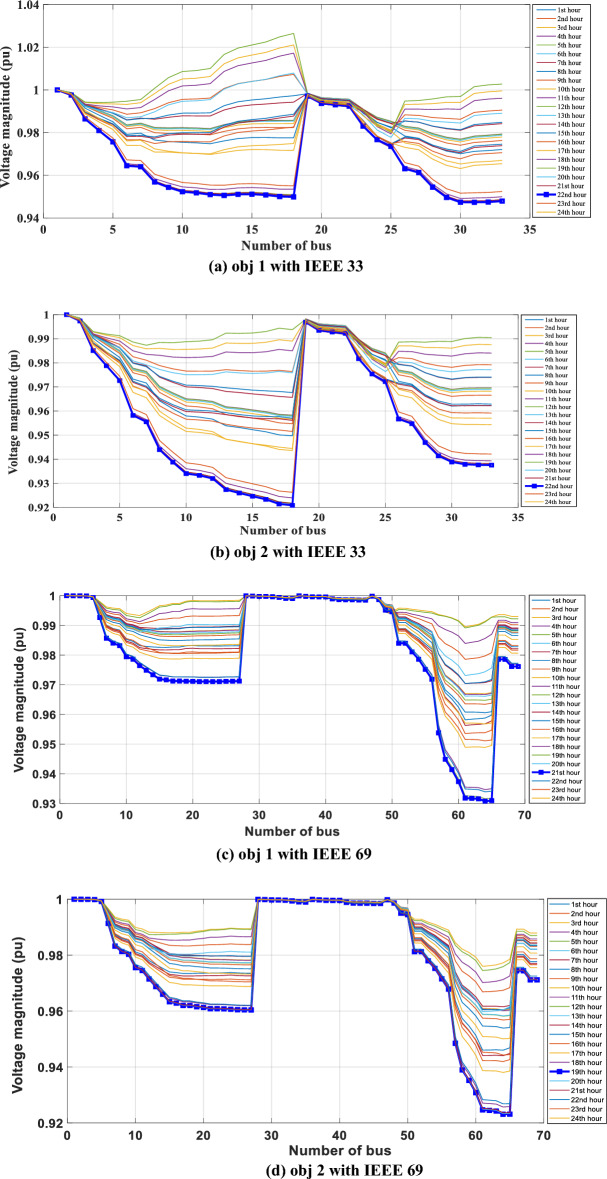
Voltage profile variation over a 24-h period for the third scenario in the two network.

Figure 9a–d showcase voltage profiles across the IEEE 33 and IEEE 69 networks, revealing how these profiles not only meet but exceed the minimum thresholds established by their respective base cases. This achievement is particularly noteworthy during peak consumption hours, where voltage stability is crucial for maintaining network reliability. For the IEEE 33 network, the voltage profiles achieve minimum levels of 0.94 pu and 0.92 pu for the primary and secondary objectives, respectively, during these critical periods. Meanwhile, the IEEE 69 network demonstrates a commendable performance with voltage levels sustaining at 0.93 pu and 0.92 pu for the primary and secondary objectives, respectively, even amidst peak demand.

These findings underscore the efficacy of the Multi-Objective Multi-Verse Optimization (MOMVO) method in optimizing the deployment of Photovoltaic and Reactive Energy Source Capacitor Banks (PVRES-CBs) within these networks. By adjusting PVRES and CB configurations, the MOMVO method not only ensures voltage levels remain above critical thresholds but also contributes to enhancing the overall stability and reliability of the power supply during peak demand periods. This successful optimization underscores the importance of advanced algorithmic approaches like MOMVO in achieving operational excellence in power distribution networks, ensuring they can reliably meet increased demand without compromising on service quality.

The study meticulously explores the application of the Multi-Objective Multi-Verse Optimization (MOMVO) algorithm for optimizing Distributed Generation (DG) and compensation devices across electrical distribution networks, offering a novel approach to addressing complex optimization challenges. Through comparative analysis, MOMVO's superior performance is highlighted against alternatives like MOJS, MOFPA, and MOLA within IEEE 33-bus and IEEE 69-bus networks. The utilization of Pareto front analysis underpins the study’s effectiveness in delineating the trade-offs between minimizing energy losses, voltage deviations, and operational costs, showcasing the algorithm's adeptness at navigating the intricate multi-objective optimization landscape. To enrich the evaluation, a deeper statistical analysis could include metrics such as convergence speed, solution diversity, and rigorous statistical significance testing, enhancing the robustness and reliability assessment of the MOMVO algorithm. Moreover, exploring the algorithm's sensitivity to varying network configurations and its performance stability across diverse scenarios would provide comprehensive insights into its applicability. Extending the analysis to real-world distribution networks, through statistical validation against actual data, would further affirm MOMVO’s practical utility and efficacy in dynamic, real-world conditions, marking a significant advancement in distribution system optimization research.

## Conclusion

This study pioneers the development of a sophisticated multi-objective optimization framework, focusing primarily on the strategic placement and sizing of photovoltaic (PV) systems and capacitor banks within electrical distribution networks. Incorporating dynamic environmental factors such as irradiance levels, temperature fluctuations, and load variations, this innovative framework aims to concurrently minimize energy losses, voltage deviations, and operational expenses. The Multi-Objective Modified Vector Optimization (MOMVO) methodology is employed to tackle the complex challenge of optimizing distribution networks enriched with renewable energy sources. The efficacy of MOMVO is demonstrated through its application on IEEE 33-bus and IEEE 69-bus test systems, simulating real-world conditions. An external memory bank facilitates the capture and storage of Pareto-optimal solutions, enabling efficient exploration of the multi-dimensional solution space. A fuzzy ranking method aids in identifying the most favorable compromise solutions.

Our findings reveal a significant reduction in daily energy losses and improvements in voltage stability across both test systems. For the IEEE 33 network, the MOMVO algorithm facilitated a substantial 47.58% decrease in daily energy loss and improved the voltage profile stability from 0.89 to 0.94 pu. Furthermore, it achieved a 36.97% reduction in the annual cost of energy losses, underscoring the substantial economic benefits. For the larger IEEE 69 network, the application of MOMVO resulted in a remarkable 50.15% reduction in energy loss and enhanced voltage profiles from 0.89 to 0.93 pu, with the annual cost of energy losses experiencing a 47.59% reduction. These numerical results not only confirm the robustness of the MOMVO algorithm in enhancing technical and economic efficiencies but also highlight the potential of advanced optimization techniques in promoting the sustainable integration of renewable energy resources into existing power infrastructures.

Comparative analysis with alternative algorithms such as the Multi-Objective Jaya Search (MOJS), Multi-Objective Lion Algorithm (MOLA), and Multi-Objective Flower Pollination Algorithm (MOFPA) underscores the superior performance of the MOMVO approach in terms of solution quality and convergence speed. This research not only extends the boundaries of current distribution system optimization practices but also lays a solid foundation for further studies aimed at more effectively integrating renewable energy sources and optimizing power systems for improved efficiency, reliability, and sustainability. Through the insights obtained, this study makes a significant contribution to the field of power system optimization, paving the way for future advancements in the intelligent integration of renewable energy and optimization techniques**.**

### Supplementary Information

Supplementary Information.

## Data Availability

The datasets used and/or analysed during the current study available from the corresponding author on reasonable request.

## Abbreviations

DGs: Distributed generation
PVRES: Photovoltaic renewable energy sources
CB: Capacitor banks
MOMVO: Multi-objective multi-verse optimization
MOFPA: Multi-objective flower pollination
MOLA: Multi-objective Lichtenberg algorithm
MOPSO: Multi-objective particle swarm optimization
PFNDMOPSO: Pareto front non-dominated multi-objective particle swarm optimization
TLBO: Teaching–learning-based optimization
V_mpp_: Voltage at the maximum power point
I_mpp_: Current at the maximum power point
(mpp): Maximum power point
Eloss: Energy losses daily
I_br_: The current flow through each distribution branch
R_br_: The resistance of each distribution branch
VDD: The voltage deviation per day
Vj: The voltage magnitude at each distribution node (j)
Closs: The yearly cost attributed to energy losse
TOC: The cost of power supplied by Photovoltaic and Reactive Energy Source Capacitor Banks
Kp: The annual cost of energy losses ($ /kwh)
Kp: The purchase cost ($ /KW)
PPV: The active power of the photovoltaic k
KQ: The purchase cost ($ /KVAR)
Qcb: The reactive power of the capacitor bank k
PPVRES, max: The maximum amount of electrical power that the PVRES can generate under optimal conditions
QCB, max: The maximum amount of reactive power that the CB can supply to the system
V_jmin_: The minimum allowable voltage at node j
V_jmax_: The maximum allowable voltage at node j
I_brmax_: The maximum allowable current for a distribution branch
Kp: The penetration limit
LMSE: Laboratory for energy systems modeling
