# Supplementary material for: A pareto strategy based on multi-objective optimal integration of distributed generation and compensation devices regarding weather and load fluctuations
Source: Sci Rep. 2024 May 7;14:10423. doi: 10.1038/s41598-024-61192-2 (PMC11074325; doi:10.1038/s41598-024-61192-2)
Supplement: Supplementary file 1 — Supplementary Information. [file 41598_2024_61192_MOESM1_ESM.docx]

**APPENDIX**

The LMSE laboratory at Biskra University holds statistical data concerning the fluctuations of PVRES, considering both irradiance and temperature across the two networks. The mean variations of PVRES are outlined in Table A.

**TABLE S1. Annual daily average variation of PVRES with irradiance and temperature for IEEE 33, IEEE 69**

| Hours | Solar Radiation (w/m^2) | Temperature |
| --- | --- | --- |
| 1  2  3  4  5  6  7  8  9  10  11  12  13  14  15  16  17  18  19  20  21  22  23  24 | 0  0  0  0  0  32  200  400  575  733  872  950  900  818  732  565  455  139  37  0  0  0  0  0 | 25  25  25  25  25  25  25  35  35  35  45  45  55  60  55  45  45  35  25  25  25  25  25  25 |
